# Avian IRF1 and IRF7 Play Overlapping and Distinct Roles in Regulating IFN-Dependent and -Independent Antiviral Responses to Duck Tembusu Virus Infection

**DOI:** 10.3390/v14071506

**Published:** 2022-07-09

**Authors:** Chengwei Xiang, Zekun Yang, Ting Xiong, Ting Wang, Jie Yang, Mei Huang, Dingxiang Liu, Ruiai Chen

**Affiliations:** 1 College of Veterinary Medicine, South China Agricultural University, Guangzhou 510642, China; xiangchengwei92@sina.cn (C.X.); zekunyang961224@163.com (Z.Y.); bearvet@163.com (T.X.); 15283860702@163.com (T.W.); iamyoungjay@163.com (J.Y.); 2 Zhaoqing Branch Center of Guangdong Laboratory for Lingnan Modern Agricultural Science and Technology, Zhaoqing 526000, China; 3 Zhaoqing Institute of Biotechnology Co., Ltd., Zhaoqing 526238, China; huangmeigz@163.com; 4 Integrative Microbiology Research Centre, South China Agricultural University, Guangzhou 510642, China

**Keywords:** duck tembusu virus, IRF1, IRF7, antiviral response, nonstructural protein NS2B

## Abstract

Avian interferon regulatory factors 1 and 7 (IRF1 and IRF7) play important roles in the host’s innate immunity against viral infection. Our previous study revealed that duck tembusu virus (DTMUV) infection of chicken fibroblasts (DF1) and duck embryo fibroblasts (DEFs) induced the expression of a variety of IFN-stimulated genes (ISGs), including VIPERIN, IFIT5, CMPK2, IRF1, and IRF7. IRF1 was further shown to play a significant role in regulating the up-expression of VIPERIN, IFIT5, and CMPK2 and inhibiting DTMUV replication. In this study, we confirm, through overexpression and knockout approaches, that both IRF1 and IRF7 inhibit DTMUV replication, mainly via regulation of type I IFN expression, as well as the induction of IRF1, VIPERIN, IFIT5, CMPK2, and MX1. In addition, IRF1 directly promoted the expression of VIPERIN and CMPK2 in an IFN-independent manner when IRF7 and type I IFN signaling were undermined. We also found that non-structural protein 2B (NS2B) of DTMUV was able to inhibit the induction of IFN-β mRNA triggered by Newcastle disease virus (NDV) infection or poly(I:C) treatment, revealing a strategy employed by DTMUV to evade host’s immunosurveillance. This study demonstrates that avian IRF7 and IRF1 play distinct roles in the regulation of type I IFN response during DTMUV infection.

## 1. Introduction

Tembusu virus, first isolated from Culex mosquito in Malaysia in 1955 [1], was found to cause an infectious disease characterized by encephalitis and growth retardation in chicks in Malaysia in 2000 [2]. Ten years later in 2010, it was confirmed to be the causative agent of an unknown duck egg-laying syndrome that broke out in the Southeastern coastal provinces in China, with a significant drop in egg production in laying ducks and neurological symptoms in infected meat ducks [3]. Subsequently, large-scale outbreaks of duck tembusu virus (DTMUV) infection occurred in major duck farming areas in the country, spreading rapidly and causing huge economic losses.

Innate immunity is considered to be the first line of host defense against virus invasion and replication. Type I interferons (IFNs) are one of the most important defense genes that bind to IFN receptors and drive the expression of more than one thousand IFN-stimulated genes (ISGs) essential for cell defense and inflammation. Among them, IFN regulatory factor 3/7 (IRF3/7) and IRF1 are essential transcription factors in the activation of type I IFN signaling. 

IRF1 was firstly identified in 1988 [4] from a nuclear extract of mouse L929 cells infected with Newcastle Disease Virus (NDV). Subsequently, it was found to be commonly expressed in human cells at a low basal level [5] and is highly sensitive to a variety of stimuli, including IFNs and pro-inflammatory nuclear factor kappa-B (NF-κB) [6,7]. IRF1 contains a DNA binding domain (DBD) consisting of 115 amino acids at the N-terminal region with five tryptophan residues [8]. The downstream of DBD is the nuclear localization signal (NLS, amino acid residue 117–141), which promotes IRF1 nuclear residency [9]. DBD is followed by the unique C-terminal IRF-associated domain (IAD2, amino acid residues 200–262) of the IRF1 subfamily, which interacts with other transcription factors to form a transcription regulatory complex [10]. 

IRF7 of all species contains a conserved N-terminal DBD and an IAD [11]. Among IRF family members, IRF7 and IRF3 play a key role in type I IFN-mediated innate immunity. However, it has been confirmed that in birds only IRF7 exists and IRF3 is absent [12,13], and IRF7 is the most critical IFN-β regulator in chickens. 

DTMUV belongs to the flavivirus family, causing duck oophoritis and encephalitis [14]. Like other flaviviruses, DTMUV is a single-stranded positive-sense RNA virus with a genome size of 10,990 bp. Its single open reading frame (ORF) encodes a unique polyprotein precursor, which is then cleaved into three structural proteins (C, prM, and E) and seven non-structural (NS) proteins (NS1, NS2A, NS2B, NS3, NS4A, NS4B, and NS5) [15,16]. Previous studies have shown that DTMUV infection can effectively trigger type I IFN signaling and downstream ISG expression [17,18]. Avian type I IFNs and ISGs, such as VIPERIN, IFIT5, CMPK2, IRF7, IRF1, Mx, and OASL, play important roles in combating DTMUV infection [19,20,21,22]. On the other hand, the virus itself develops a variety of strategies to countermeasure the innate immune response. For example, NS2B was shown to cleave the duck stimulant (STING) gene, leading to the reduction in the IRF7-dependent IFN induction of downstream molecules [23,24]. DTMUV NS2A can competitively bind to STING with TBK1, thus reduce TBK1 phosphorylation [25]. However, the molecular mechanism of how IRF1 and IRF7 coordinately regulate DTMUV replication remains unclear.

In this study, we examined the regulatory roles of IRF1, IRF7, and IFN-β in DTMUV replication and the expression profiles of infection-induced genes by constructing two knockout cell lines, KO IRF7 and KO IFNAR1, using CRISPR/Cas9 technology. Our results revealed that avian IRF1 and IRF7 may inhibit the replication of DTMUV by regulating the expression of IFN-β and VIPERIN, as well as a number of other ISGs via IFN-dependent and/or -independent mechanisms. Furthermore, DTMUV NS2B protein alone was confirmed to be able to inhibit the induction of IFN-β, IRF1, and IRF7 and the downstream genes VIPERIN, IFIT5, and CMPK2 in the infected cells.

## 2. Materials and Methods

### 2.1. Antibodies, Cells, and Viruses

Antibodies used in this study were purchased from the following companies: anti-Flag (#2044) and -actin (#4967) antibodies from Cell Signaling Technology (Danvers, MA, USA); anti-MYC antibody from Transgen (Beijing, China); goat anti-rabbit IgG and goat anti-mouse IgG conjugated with FITC (fluorescein isothiocyanate) from Li-COR Biosciences (Lincoln, NE, USA). Monoclonal antibody against the DTMUV E protein was described in a previous study [26]. 

Chicken fibroblast cell line was cultivated in DMEM (Carlsbad, CA, USA) supplemented with 100 U/mL penicillin, 100 μg/mL streptomycin, and 1% foetal bovine serum (FBS) (Carlsbad, CA, USA). All cells were grown in a 37 °C incubator supplied with 5% CO_2_. Preparation of duck embryonic fibroblast cells (DEFs) from 13-day-old SPF duck embryos was carried out as described [27]. 

Cells were grown on a 10 cm diameter plate and were rinsed twice with serum-free media before undergoing infection with DTMUV at a multiplicity of infection (MOI) of 1 in serum-free medium. In mock controls, identical amounts of UV-inactivated DTMUV were utilized. Viral infection was carried out at a biosafety level 2 laboratory at Veterinary Medicine college, South China Agricultural University.

Infection of cells with DTMUV strain QY17 (GenBank Accession No. MT447092) and UV-inactivation of the virus were carried out essentially, as previously described [27]. Cells were washed twice with serum-free medium and either infected with DTMUV at a multiplicity of infection (MOI) of approximately 1 or incubated with an equal volume of UV-inactivated DTMUV in serum-free medium. After 2 h of incubation, cells were washed twice with serum-free medium and incubated at 37 °C before harvesting.

### 2.2. Construction of an IFNAR1-Knockout DF1 Cell Clone (KO-IFNAR1) and an IRF7-Knockout DF1 Cell Clone (KO-IRF7) Using the CRISPR/Cas9 Technique

KO-IFNAR1 was selected from DF1 cells transfected with pX459-IFNAR1 with 5 g/mL puromycin, and KO-IRF7 was constructed in the same way by transfection of DF1 cells with pX459-IRF7. Sequencing analysis of the knockout clones confirmed deletions of two bases at positions T_232_ and A_233_ in the IFNAR1^−/−^ cell line (Figure S1A) and at positions C_2851_ and G_2852_ in IRF7^−/−^ cell line (Figure S1B). Evaluation of the growth characteristics of both KO cell lines demonstrated that knockout of either gene did not cause noticeable abnormalities (Figure S2). 

#### 2.2.1. RNA Extraction, Library Preparation, and Illumina HiSeq-X-Ten Sequencing

Duck embryo fibroblasts and chicken fibroblasts were infected with DTMUV or treated with UV-DTMUV for 24 h, respectively, and collected in triplicate. Total RNA was extracted using TRIzol® reagent according to the manufacturer’s instructions (Invitrogen, Carlsbad, CA, USA), and the genomic DNA was removed by digestion with DNase I (TaKara, Kusatsu, Japan). RNA quality was determined by using a 2100 Bioanalyzer (Agilent, Santa Clara, CA, USA) and quantified using a ND-2000 (NanoDrop Technologies, Wilmington, DE, USA). Only high-quality RNA samples (OD260/280 = 1.8∼2.2, OD260/230 ≥ 2.0, RIN ≥ 6.5, 28S:18S ≥ 1.0, >10 μg) were used for sequencing and library construction.

RNA-seq transcriptome libraries were prepared using the TruSeq™ RNA Sample Prep Kit from Illumina (San Diego, CA, USA), following the manufacturer’s instructions. Messenger RNA was isolated by oligo (dT) beads and then fragmented with fragmentation buffer. Double-stranded cDNA was synthesized by using the SuperScript double-stranded cDNA synthesis kit (Invitrogen, Carlsbad, CA, USA) with random hexamer primers (Illumina). The synthesized cDNAs were then subjected to end repair, phosphorylation, and “A” base additions according to Illumina’s library construction protocol. Libraries were size-selected, and only 200–300 bp cDNA fragments isolated on 2% low-range ultra-agarose were amplified by 15 cycles using Phusion DNA polymerase (NEB, Ipswich, MA, USA).

#### 2.2.2. Read Mapping

Raw paired-end reads were trimmed and quality-controlled by SeqPrep (https://github.com/jstjohn/SeqPrep, accessed on 6 June 2021) and Sickle (https://github.com/najoshi/sickle, accessed on 6 June 2021) with default parameters. Clean reads were individually aligned to the reference genome using the orientation mode of TopHat software (http://ccb.jhu.edu/software/tophat/index.shtml, accessed on 6 June 2021) [28]. The mapping criteria for bowtie are as follows: Sequencing reads should uniquely match the genome, allowing up to 2 mismatches with no insertions or deletions. Then the gene region was expanded according to the locus depth to obtain the operon. In addition, the entire genome was divided into multiple 15 kbp windows sharing 5 kbp. Newly transcribed regions were defined as two or more consecutive windows without overlapping regions, where each window had at least two reads mapped in the same orientation.

#### 2.2.3. Differential Expression Analysis and Feature Enrichment

To identify differentially expressed genes (DEGs) between DTMUV-infected and mock-treated DEF and DF1 samples, the expression level of each transcript was mapped according to the number of fragments per million exons per kilobase (FRKM) method computational. RSEM [29] was used to quantify gene abundance. The R statistical package Empirical Analysis of Digital Gene Expression in R (EdgeR) [30] was used for differential expression analysis.

### 2.3. RNA Extraction and RT-qPCR Analysis

Total RNAs were extracted using the TRIzol reagent (Invitrogen, CA, USA) and reverse-transcribed with PrimeScriptTM RT Master Mix (Takara, Kusatsu, Japan), and the induction levels of relevant transcripts were quantified by Quantitative PCR (qPCR) using the SYBR® Premix Ex TaqTM II Kid with a QuantStudio 3 Real-Time PCR System (Applied Biosystems, Waltham, MA, USA). The procedure consists of 3 min of enzyme activation at 50 °C, 3 min of primary denaturation at 95 °C, 40 denaturing cycles for 5 s at 95 °C, and annealing and extension for 30 s at 60 °C, with fluorescence acquisition at each cycle end. The acquired data were expressed as cycle threshold (CT) values. After normalizing to the internal β-actin value, the 2^−^^ΔΔCT^ technique was utilized for estimating the relative abundance of each transcript. 

The gene specific primers for qPCR are as follows: chGAPDH, 5′-GCCATCA CAGCCACACAGA-3′ and 5′-TTTCCCCA CAG CCTTAGCA-3′; chVIPERIN, 5′-TCGTTCTGCCTCTGCTCTCCTG-3′ and 5′-TTGTAGTTGCACTGCCTGG TGAAG-3′; chIFIT5, 5′-CACCAGCT AGGACTCTGCTACCG-3′ and 5′-CCT CCGCATACATCCTTGCCAAG-3′; chCMPK2, 5′-ATCGGTGCTGGACATCCT GGAG-3′ and 5′-GCAAGCTGG CGGAGACCTTAAC-3′; chIRF1, 5′-AAGG AGCAGGACGGCGAGATC-3′ and 5′-ACGGTGT CCAGCCAGGAGAAG-3′; chIRF7, 5′-ACACTCCCACAG ACAGTACTGA-3′ and 5′-TGTGTGTGCCCA CAGGGTTG-3′; chIFN-β, 5′-A CACTCCCACAG ACAGTACTGA-3′ and 5′-TG TGTGTGCCCACAGGGTTG -3′; chMX1, 5′-CTGCGGACAAGCCATA GAA -3′ and 5′-GCACCCCAAAAACTCCTACA-3′; DTMUV-e, 5′-CGCTG AGATGGAGG ATTATGG-3′ and 5′-ACTGATTTTTGGTGGCGTG -3′.

### 2.4. Plasmid Construction

Expression plasmids XJ40-FLAG-chIRF7, XJ40-MYC-duIRF1, XJ40-MYC-NS1, XJ40-MYC-NS2A, XJ40-MYC-NS2B, XJ40-MYC-NS3, XJ40-MYC-NS4A, and XJ40-MYC-duNS4B were generated by introducing the relevant PCR products to a pXJ40-based plasmid. The PCR products were amplified with primer pairs: Chicken IRF7, 5′-GGATCCATGGCAGCACTGGACA-3′, and 5′-CTCGAGTCAGTCTGTCTG CATGTGGTA-3′, Duck IRF1, 5′-GGATCCATGCCCGTCTCCAG-3′ and 5′-CTCGA GTTACAACCACAGGAGA-3′, NS1, 5′-GGATCCAAGCTTGACACGGGG-3′ and 5′-CTGGTACCTTAAGCCATGACCT-3′, NS2A, 5′-GGATCCAAGCTTTTTCAAGGG GT-3′ and 5′-GGTACCTTATCTCCGTGTCACTG-3′, NS2B, 5′-GGATCCAAGCT TGGGTGGCC-3′ and 5′-GGTACCTTATCGTTGTTTTGCCTTGG-3′, NS3, 5-’GGATCC AAGCTTGGAGGAGTCa-3′ and 5′-GGTACCTTATCTCTTTCCACTCGC-3′, NS4A, 5′-GGATCCAAGCTTTCAGCGATAGGG-3′ and 5′-GGTACCTTATCTCTGTCTCTCT G-3′, NS4B, 5′-GGATCCAAGCTTAATGAAATGGGT-3′ and 5′-GGTACCTTACCG ACGCAAGG-3′.

Plasmid X459-IRF7, coding for the small guide RNA targeting chicken IRF7, was produced by introducing two complementary oligonucleotides into pX459. The sequences of the two oligonucleotides are 5′-*CACCg*GGTCGTCGTTGCACTT GGAG-3′ and 5′-*AAAC*CTCCAAGTGCAACGACGACC*c*-3′, with Bbs1 ends indicated in italic. Plasmid X459-IFNAR1, coding for the small guide RNA targeting chicken IFNAR1, was produced by introducing two complementary oligonucleotides (5′-*CACCg*ACCCTAATGTGGAACTACA C-3′ and 5′-*AAA*TGGGATTACACCT TGATGTG*c*-3′, with Bbs1 ends indicated in italic), into pX459. The guide RNA was designed using the online program (www.e-crisp.org/E-CRISP) (accessed on 6 June 2021).

### 2.5. Transfection, SDS-PAGE, and Western Blot Analysis 

Transfection of plasmid DNA into cells was performed with the TransIntroTM EL Transfection Reagent (Transgen, Beijing, China), as previously described [26]. At 24 h post-transfection, cells were infected with DTMUV at an MOI of 1 or mock-treated with UV-DTMUV and cultured in FBS-free media until harvest for the extraction of proteins and/or RNA at the indicated time post-infection.

Proteins in the total cell lysates were separated by SDS-PAGE and transferred to a nitrocellulose membrane. The membrane was probed by incubation with primary antibodies and a secondary antibody conjugated with fluorescein isothiocyanate, and protein bands were detected with an Azure c600 imager. Densitometric quantification of band density was performed using the NIH programme Image J (https://imagej.nih.gov/ij/) (accessed on 18 October 2021). Each experiment was performed three times with comparable findings, and a single typical result is displayed.

### 2.6. Statistical Analysis

Comparisons between indicated samples and respective controls were conducted with one-way ANOVA. *p*-values were utilized for representing significance levels in all figures (ns, non-significant; * *p* < 0.05; ** *p* < 0.01; *** *p* < 0.0001).

## 3. Results

### 3.1. Upregulation of IRF7 and IRF1 in DTMUV-Infected DEFs and DF1 Cells as Revealed by Transcriptomic Analysis 

**Figure 1:**
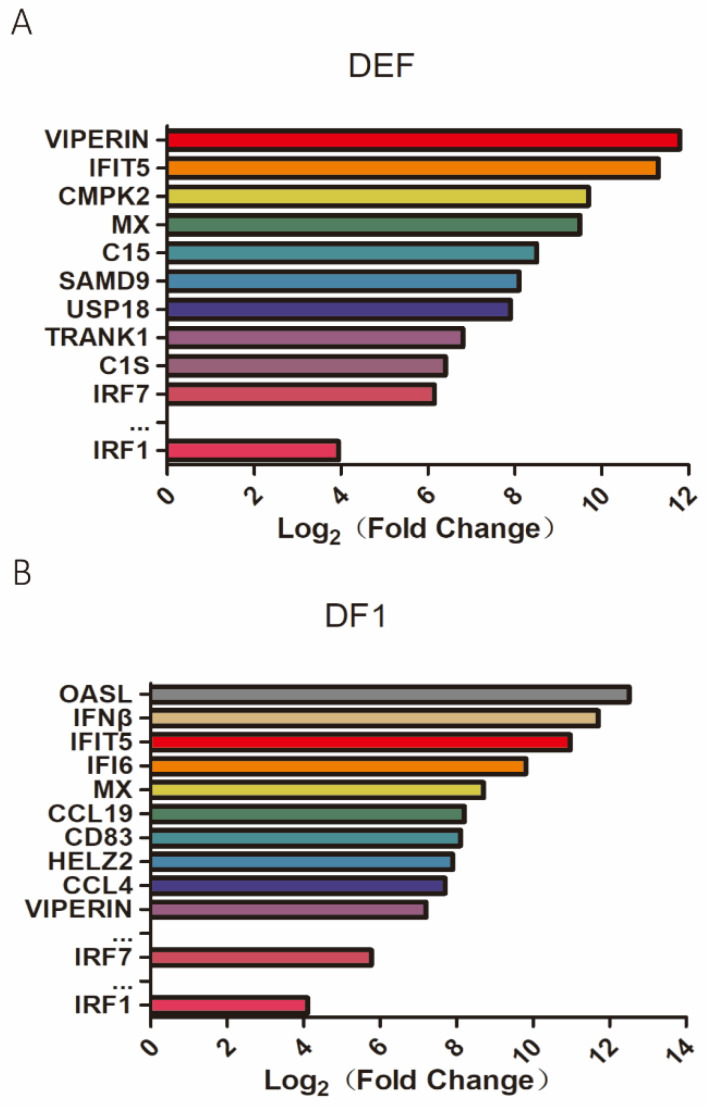
Transcriptomic analysis of differential gene expression in DEF and DF1 cells infected with DTMUV. The duck endoblastomal fibral cells (DEF) and the chicken stable cell line DF1 were infected with DTMUV at an MOI of 1 for 24 h. Total RNAs were extracted for transcriptomic analysis. The top induced genes in DTMUV-infected DEF are shown in (**A**) and DTMUV-infected DF1 cells are shown in (**B**).

The differential regulation of host gene expression in DEFs and DF1 cells infected with DTMUV QY17 or UV-DTMUV-treated at 24 hpi were analyzed by transcriptomic analysis. After normalization to the internal GAPDH transcript from mock-treated and infected cells, the ratio of each transcript was computed and shown within the *p*-value< 0.05 and |log_2_ (fold change)| > 1 thresholds. The 10 genes with the highest expression within the elevated genes in the complete transcriptome were VIPERIN, CMPK2, IFIT5, MX, SAMD9, PROTEIN C15, TRANK1, USP18, C1S, and IRF7 in DEFs, and OASL, IFNβ, IFIT5, IFI6, MX, CCL19, CD83, HELZ2, CCL4, and VIPERIN in DF1 cells (Figure 1). In addition, IRF7 and IRF1 were also significantly up-regulated in both cell types (Figure 1). qPCR results also confirmed the accuracy of transcriptome data (Figure S7). 

These differentially expressed genes in the four representative immune-relevant pathways, Toll-like receptors (TLRs), Receptor-Like Receptors (RLRs), Natural Killer Receptors (NLRs), and Hepatitis Signaling Pathways were further analyzed. Table S1 lists the 10 most up-regulated genes in each pathway, as denoted by the Log2(FC) value (Fold Change). It was also noted that in both DEFs and DF1 cells infected with DTMUV, the expression of IRF7 and IRF1 was significantly up-regulated, especially the IRF7. These findings imply that IRF7 and IRF1 may be critical regulators of DTMUV replication in DEFs and DF1 cells. Since DTMUV infection of DF1 and DEF cells significantly induces the expression of VIPERIN, IFIT5, and CMPK2, these genes may be common antiviral genes against DTMUV infection. Furthermore, our published paper has proved the roles of VIPERIN, IFIT5, and CMPK2 in inhibiting DTMUV replication [22]. These genes were therefore chosen for further investigation in this study.

### 3.2. Suppression of DTMUV Replication in DF1 Cells by IRF7

**Figure 2:**
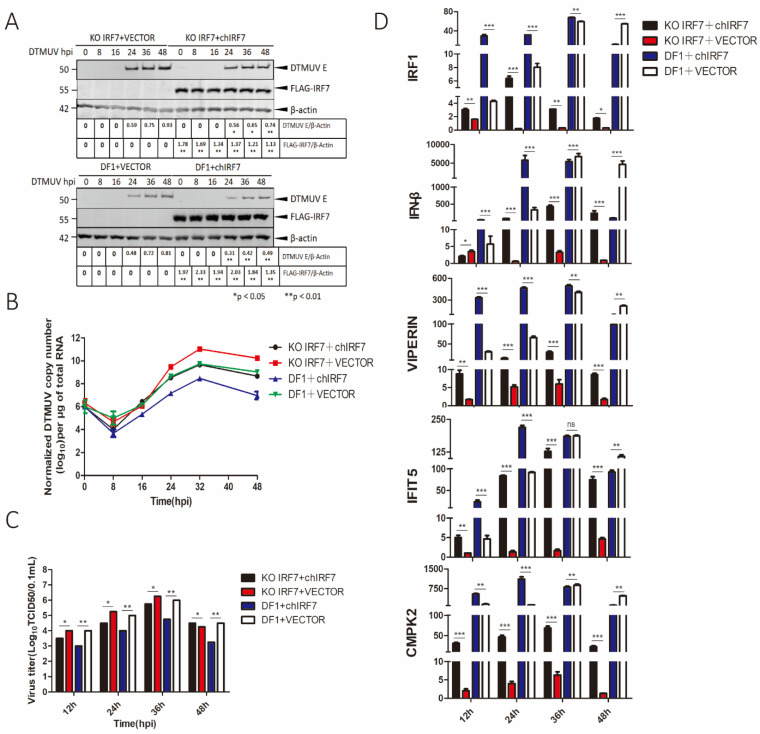
Effects of IRF7-overexpression and -knockout on DTMUV replication and upregulation of IRF1, VIPERIN, IFN-β, IFIT5, and CMPK2. (**A**) Wild type (DF1) and IRF7-knockout (KO IRF7) cells were transfected with a Flag-tagged chicken IRF7 and infected with DTMUV at an MOI of approximately 2. Cells were harvested at 0, 8, 16, 24, 36, and 48 hpi, respectively, and total cell lysats were prepared in parallel. The chiIRF7 expression levels and the replication of DTMUV were assessed by Weston Blot with anti-Flag and anti-DTMUV antibodies. β-actin was detected for a loading control. (**B**) The genomic RNA levels of DTMUV in DF1 and KO IRF7 cells, as in (**A**), were determined by RT-qPCR and presented. (**C**) Viral titers were determined by TCID50 from the extracellular fluids of DF1 and KO IRF7 cells harvested, as in (**A**). Error bar represents the standard error of three replicate experiments. *T* tests were performed between the IRF7 overexpressed cells and the empty vector transfected cells in DF1 (blue and white) and KO IRF7 (red and black) cells. (**D**) The mRNA levels of IRF1, IFN-β, VIPERN, IFIT5, and CMPK2 from the cells harvested as in (**A**) were detected by RT-qPCR. T tests were performed between the IRF7 overexpressed cells and the empty vector transfected cells in DF1 (blue and white) and KO IRF7 (red and black) cells. (ns, nonsignificant; * *p* < 0.05; ** *p* < 0.01; *** *p* < 0.001).

As IRF7 was significantly up-regulated in DTMUV-infected cells, its functional significance and underlying mechanisms in regulation of DTMUV replication were studied by knockout and overexpression of IRF7, respectively, in DF1 cells. Stable knockout cell clone (KO IRF7) was obtained by knockout of IRF7 in DF1 cells using CRISPR-cas9, and chicken IRF7 (chIRF7) was used in the overexpression experiments. KO IRF7 and WT DF1 cells transiently expressing the FLAG-tagged chIRF7 were infected with DTMUV and harvested at specified time points. Cells transfected with an empty vector were used as a control. Overexpression of chIRF7 was confirmed by Western blot with anti-FLAG antibody (Figure 2A). Viral particles released to the culture media were titrated with TCID50 assay and the levels of viral RNA in the infected cells were determined by RT-qPCR. RT-qPCR results shown in Figure 2B confirmed that the viral RNA level in WT cells transfected with chIRF7 (blue) was reduced by 32- to100-fold at 12 and 48 hpi, while it increased by 10- to 100-fold in KO IRF7 cells (red) at 24 and 48 hpi, compared with that in the WT control group (green). Overexpression of IRF7 in KO IRF7 (black) reduced the viral RNA level by 20- to 100-fold at 24 and 48 hpi, compared with the control group (red) (Figure 2B). Viral titers in the culture media collected from KO IRF7 (red) were 3- to 4-fold higher at 24 and 36 hpi than in WT cells, while WT cells transfected with chIRF7 (blue) were 8- to 10-fold lower at 12 and 48 hpi than in control cells transfected with the vector, and in KO IRF7 cells overexpressing IRF7 (black), they were 3- to 6-fold lower at 24 and 36 hpi than in KO IRF7 control cells (Figure 2C). The slightly increased viral titers observed in the KO IRF7 group may reflect the limited replication efficiency of DTMUV in DF1 cells. These results confirm that IRF7 may play an important role in restriction of DTMUV replication in DF1 cells.

The function of IRF7 as a main regulator in the induction of IRF1, VIPERIN, IFIT5, CMPK2, and MX1 in DTMUV-infected cells was then studied by checking the effect of IRF7 overexpression on the induction of IRF1, IFN-β, VIPERIN, IFIT5, CMPK2, and MX1 in DTMUV-infected DF1 and knockout cells. As shown in Figure 2D, Figures S3 and S9, the mRNA levels of IRF7, IRF1, IFN-β, VIPERIN, IFIT5, CMPK2, and MX1 in IRF7-overexpressing DF1 cells (blue) were 60-to130-fold, 20- to 25-fold, 90- to 5800-fold, 270- to 440-fold, 25- to 140-fold, 400- to 600-fold, and 10- to 2000-fold, respectively induced, compared with the control group transfected with an empty vector at 12–24 hpi. DTMUV infection-induced expression of IRF1, IFN-β, VIPERIN, IFIT5 CMPK2, and MX1 (red) was much reduced in KO IRF7 cells, compared with that in WT cells (Figure 2D and Figure S9A). Notably, the induction of IRF1and IFNβ in IRF7-overexpressing KO IRF7 cells (black) was 2- to 7-fold, and 100- to 400-fold increased, compared with that in the KO IRF7 control group (red) at 24–36 hpi (Figure 2D). The ELISA results also further verified the expression level of IFN-β (Figure S8A). It is worth noting that overexpression of IRF7 in KO IRF7 cells almost completely restored the expression of IFIT5, but the induction of IRF1, IFN-β, CMPK2, VIPERIN, and MX1 was only partially restored, suggesting the involvement of other regulator(s). 

### 3.3. Differential Roles of Type I IFN Signaling in IRF7-Mediated Upregulation of IRF1, IFN-β, VIPERIN, IFIT5, CMPK2, and MX1 in DTMUV-Infected Cells 

**Figure 3:**
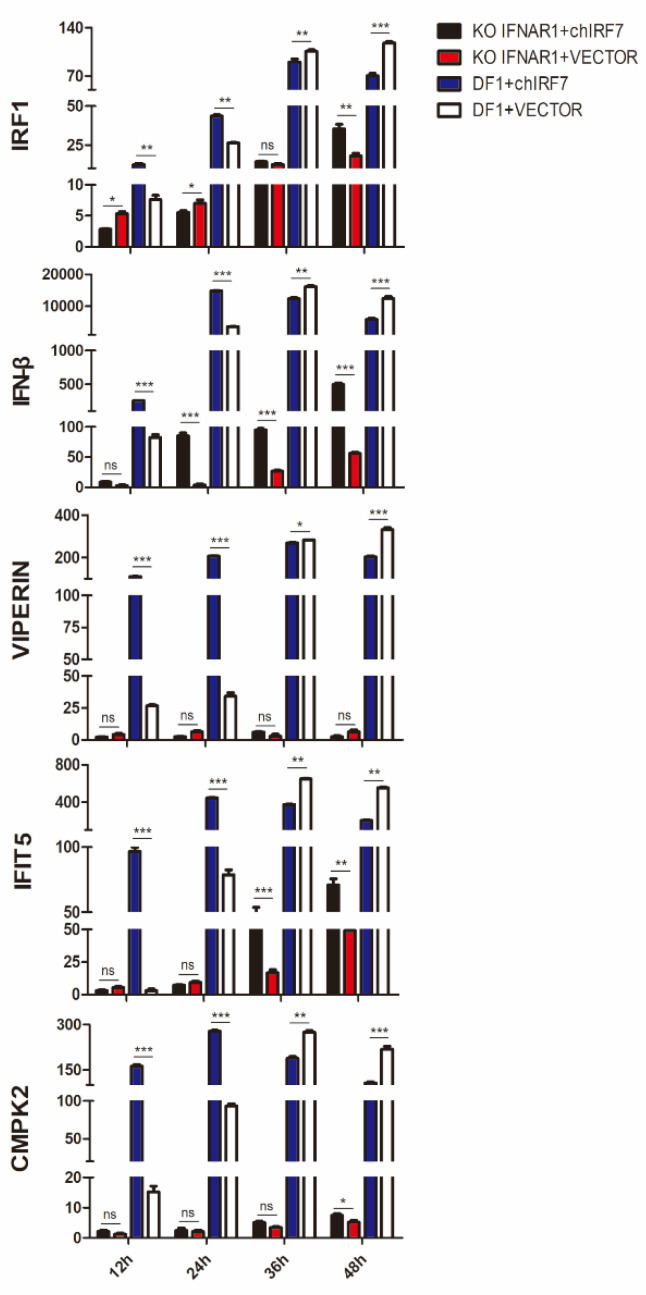
Effects of IRF7-overexpression on the upregulation of IFN-β, IFIT5, VIPERIN, CMPK2, and IRF1 induced by DTMUV in IFNAR1-knockout DF1 cell (KO IFNAR1)**.** DF1 and KO IFNAR1 cells were transfected with a Flag-tagged chicken IRF7 and infected with DTMUV at an MOI of approximately 2. Cells were harvested at 12, 24, 36, and 48 hpi, respectively. Total RNAs were extracted, and the induction levels of IFN-β, VIPERIN, IFIT5, CMPK2, and IRF1 were determined by RT-qPCR and presented. Error bar represents the standard error of three replicate experiments. T tests were performed between the samples with or without IRF7-overexpression in KO IFNAR1 (red and black) and DF1 cell (blue and slash). (ns, nonsignificant; * *p* < 0.05; ** *p* < 0.01; *** *p* < 0.001).

The mechanisms underlying the IRF7-mediated induction of IRF1, IFN-β, VIPERIN, IFIT5, CMPK2, and MX1 were then studied by overexpression of IRF7 in IFNAR1-knockout cells (KO IFNAR1) infected with DTMUV. As shown in Figure 3 and Figure S9B, the induction of these five genes was severely suppressed in KO IFNAR1 cells at 12–48 h post-DTMUV infection. Among them, the induction of VIPERIN and CMPK2 was almost completely blocked in the knockout cells, and overexpression of IRF7 did not restore their induction level (Figure 3), suggesting that type I IFN signaling is essential for the induction of these two genes. Overexpression of IRF7 in KO IFNAR1 cells infected with DTMUV partially restored the expression of IFN-β at 12–48 hpi, IFIT5 at 36–48 hpi, IRF1 at 48 hpi (Figure 3), and MX1 at 12–48 hpi (Figure S9B), indicating a type I IFN-independent role of IRF7 in the induction of these genes during DTMUV infection. The ELISA results also further verified the expression level of IFN-β (Figure S8B). 

### 3.4. Modulation of DTMUV Replication and the Expression of IFN-β, VIPERIN, IFIT5, CMPK2, and MX1 by IRF1 in a Type I IFN-Dependent or -Independent Manner

**Figure 4:**
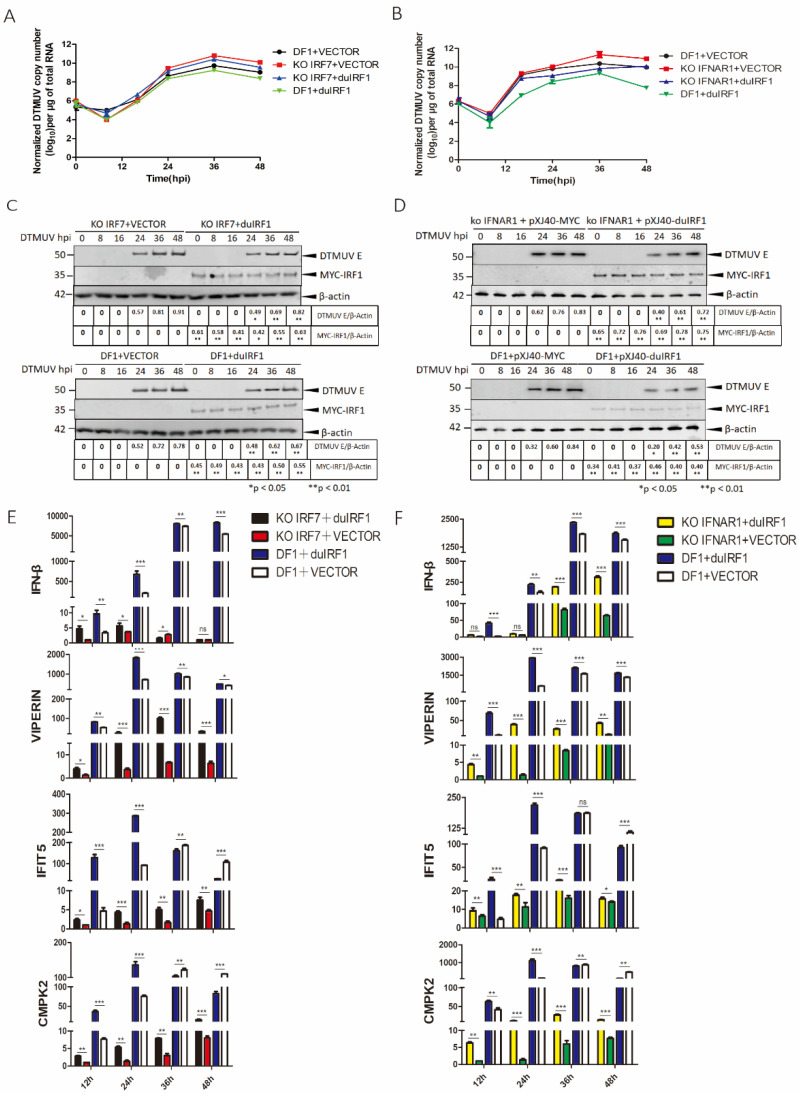
Inhibition of DTMUV replication and regulation of the expression of IFN-β, VIPERIN, IFIT5, and CMPK2 induced by overexpression of duck IRF1 in KO IRF7 and KO IFNAR1 cells. (**A**) DF1 and KO IRF7 cells were transfected with a Myc-tagged duIRF1, infected with DTMUV at an MIO of approximately 2, and harvested at 12, 24, 36, and 48 hpi, respectively. Total RNAs were extracted, and the copy numbers of the viral genomic RNA were determined by RT-qPCR and presented. (**B**) DF1 and KO IFNAR1 cells were transfected with a Myc-tagged duIRF1, infected with DTMUV at an MIO of approximately 2, and harvested at 12, 24, 36, and 48 hpi, respectively. Total RNAs were extracted, and the copy numbers of the viral genomic RNA were determined by RT-qPCR and presented. (**C**) DF1 and KO IRF7 cells were transfected with a Myc-tagged duIRF1, infected with DTMUV at an MIO of approximately 2, and harvested at 12, 24, 36, and 48 hpi, respectively. Total cell lysats were prepared, and the duIRF1 expression levels and the replication of DTMUV were assessed by Weston Blot with anti-Myc and anti-DTMUV antibodies. β-actin was detected for a loading control. The relative level of E protein was determined after normalization to β-actin and shown at the bottom. (**D**) DF1 and KO IFNAR1 cells were transfected with a Myc-tagged duIRF1, infected with DTMUV at an MIO of approximately 2, and harvested at 12, 24, 36, and 48 hpi, respectively. Total cell lysats were prepared, and the duIRF1 expression levels and the replication of DTMUV were assessed by Weston Blot with anti-Myc and anti-DTMUV antibodies. β-actin was detected for a loading control. The relative level of E protein was determined after normalization to β-actin and shown at the bottom. (**E**) DF1 and KO IRF7 cells were transfected with a Myc-tagged duIRF1, infected with DTMUV at an MIO of approximately 2, and harvested at 12, 24, 36, and 48 hpi, respectively. Total RNAs were extracted, and the induction levels of IFN-β, VIPERIN, IFIT5, and CMPK2 were determined by RT-qPCR and presented. Error bar represents the standard error of three replicate experiments. T tests were performed between the IRF1-overexpressing cells and vector transfected cells in DF1 (blue and slash) and KO IRF7 (red and black). (**F**) DF1 and KO IFNAR1 cells were transfected with a Myc-tagged duIRF1, infected with DTMUV at an MIO of approximately 2, and harvested at 12, 24, 36, and 48 hpi, respectively. Total RNAs were extracted, and the induction levels of IFN-β, VIPERIN, IFIT5, and CMPK2 were determined by RT-qPCR and presented. Error bar represents the standard error of three replicate experiments. T tests were performed between IRF1-overexpressing cells, vector-transfected cells in DF1 (blue and slash), and KO IFNAR1 cells (yellow and green). (ns, nonsignificant; * *p* < 0.05; ** *p* < 0.01; *** *p* < 0.001).

IRF1 was previously shown to play a restriction role in DTMUV replication [22]. The underlying mechanisms were studied by overexpressing duck IRF1 (duIRF1) in DTMUV-infected KO IRF7 (Figure 4A) and KO IFNAR1 (Figure 4B) cells. Viral RNA copy numbers were determined at indicated time points, and viral E protein and the overexpressed duIRF1 protein were confirmed by Western blot (Figure 4C,D). Overexpression of duIRF1 in WT DF1 (green) resulted in a slight (0.5- to 0.8-fold) reduction in the viral RNA copy number (Figure 4A,B), compared with the control cells transfected with an empty vector only (black). Overexpression of IRF1 in either KO IRF7 or KO IRFAR1 cells also resulted in a 0.5- to 1-fold reduction in viral RNA copy number (Figure 4A,B), indicating that IRF1 may regulate DTMUV replication in the absence of IRF7 and type I IFN signaling. The ELISA results also further verified the expression level of IFN-β (Figure S8C). Western blot analysis showed similar results. The fact that IRF1 plays a more dominant inhibitory role in DTMUV-infected KO IFNAR1 cells points to a type I IFN-independent function of the protein in the regulation of DTMUV replication. 

The regulatory role of IRF1 in the induction of IFN-β, VIPERIN, IFIT5, CMPK2, and MX1 in DTMUV-infected cells was then studied by overexpression of duIRF1 in WT, KO IRF7, or KO IFNAR1 cells during DTMUV infection. The results showed that overexpression of duIRF1 significantly increased the expression of VIPERIN, IFIT5, CMPK2, and MX1 in WT DF1 (blue), compared with the empty vector control (white) (Figure 4E,F and Figure S9). The induction of VIPERIN, IFIT5, CMPK2, and MX1 in WT DF1 cells was detected at early time points, mainly at 12–24 hpi (Figure 4E,F and Figure S9). Overexpression of IRF1 in KO IRF7 cells partially restored the induction of VIPERIN, IFIT5, and CMPK2 (Figure 4E), but only minimal induction of IFN-β and MX1 was detected (Figure 4E and Figure S9C). Similar effects were observed in KO IFNAR1 cells (Figure 4F and Figure S9). The induction of VIPERIN, IFIT5, and CMPK2 was partially restored, but only minimal induction of IFN-β and MX1 was observed (Figure 4F and Figure S9D). The ELISA results also further verified the expression level of IFN-β (Figure S8D). Attempts to construct an IRF1-knockout cell line was not succussed, but knockdown of IRF1, as shown in our previous studies, can partially inhibit the induction of VIPERIN, IFIT5, and CMPK2, especially VIPERIN [22]. Taken together, IRF1 could function as a transcription activator for VIPERIN, IFIT5, and CMPK2 during DTMUV infection. 

### 3.5. Suppression of Poly(I:C)-Induced IFN-β Expression by DTMUV NS2B Protein 

**Figure 5:**
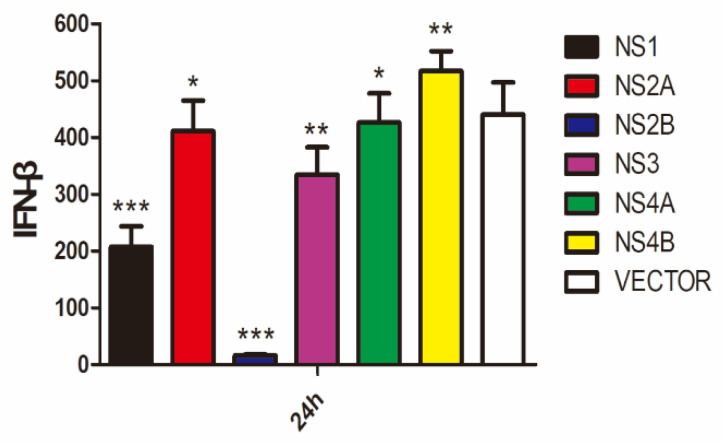
Inhibition of poly(I:C)-induced IFN-β expression by DTMUV NS2B. DF1 cells were transfected with plasmids expressing Myc-tagged DTMUV NS1, NS2A, NS2B, NS3, NS4A, or NS4B, respectively, and treated with 1μg/mL poly(I:C). Cells were harvested at 24 h post-transfection, and the protein expression levels of NS1, NS2A, NS2B, NS3, NS4A, and NS4B in poly(I:C)-treated cells were determined by Western blot with anti-Myc serum. IFN-β induction at the mRNA level from above samples was determined by RT-qPCR and presented. Error bar represents the standard error of three replicate experiments. T tests were performed by comparing the vector-transfected sample (white) to samples transfected with each of the viral gene. (ns, nonsignificant; * *p* < 0.05; ** *p* < 0.01; *** *p* < 0.001).

The strategies utilized by DTMUV to evade type I IFN signaling were then studied by screening DTMUV NSs for their IFN antagonism roles. Plasmids expressing Myc-tagged NS1, NS2A, NS2B, NS3, NS4A, and NS4B were constructed and used to transfect DF1 cells, followed by stimulation with poly(I:C). The overexpressed viral proteins were analyzed by Western blotting with an anti-Myc antibody, the induced IFN-β levels were detected by RT-qPCR, and the impact of the individual viral proteins on IFN-β induction was analyzed by comparing with the control transfected with an empty vector. As shown in Figure S5, the six viral proteins were efficiently expressed in the transfected DF1 cells. Treatment with poly(I:C) induced IFN-β expression efficiently at 24 h post treatment (Figure 5). Among the six overexpressed proteins, only NS2B showed significant inhibition of IFN-β induction. The ELISA results also further verified the expression level of IFN-β (Figure S8E). Compared with the control, a 5-fold reduction at the IFN-β mRNA level was detected in cells overexpressing NS2B (Figure 5). This result suggests that DTMUV NS2B may function as a type I IFN antagonist. 

### 3.6. Suppression of the Induction of IRF7, IRF1, VIPERIN, IFIT5, CMPK2, and IFN-β in NDV-Infected DF1 Cells by NS2B

**Figure 6:**
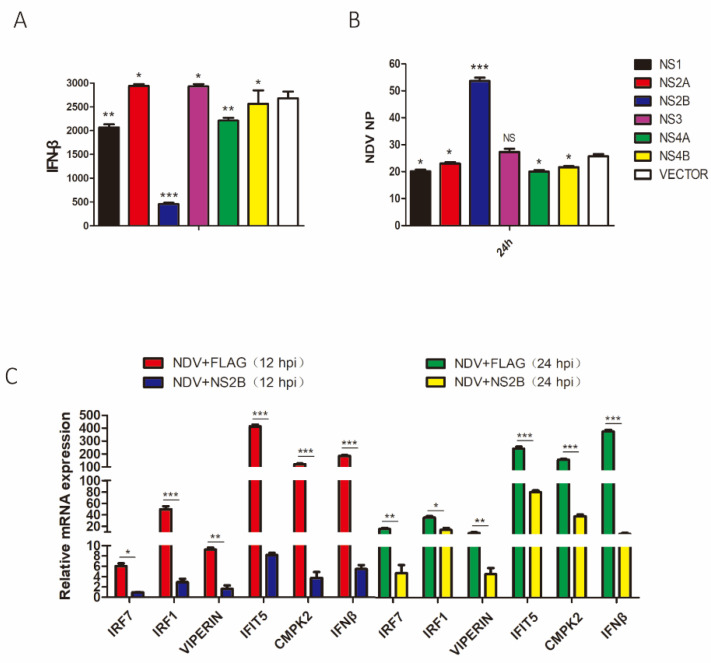
The effects of DTMUV NS2B-overexpression on NDV replication and the induction of IFN-β, as well as IFN-reducible genes. (**A**) DF1 cells were transfected with plasmids expressing Myc-tagged DTMUV NS1, NS2A, NS2B, NS3, NS4A, or NS4B, respectively, and infected with NDV at an MOI of 1. Cells were harvested at 24 h post-infection of NDV, and the protein expression levels of NS1, NS2A, NS2B, NS3, NS4A, and NS4B in poly(I:C)-treated cells were determined by Western blot with anti-Myc serum. (**B**) IFN-β induction at the mRNA level from above samples was determined by RT-qPCR and presented. Error bar represents the standard error of three replicate experiments. T tests were performed by comparing the vector-transfected sample (white) to samples transfected with each of the viral gene. (**C**) NDV copy numbers in above samples were determined and presented. Error bar represents the standard error of three replicate experiments. T tests were performed by the vector transfected sample (white) to each of the viral gene transfected samples. DF1 cells were transfected with a plasmid expressing Flag-tagged DTMUV NS2B and infected with NDV at an MOI of 1. Cells were harvested at 12 and 24 hpi, and the expression of IRF7, IRF1 VIPERIN, IFIT5, CMPK2, and IFN-β at the mRNA level was determined by RT-qPCR and presented. Error bar represents the standard error of three replicate experiments. T tests were performed between samples transfected with Flag-NS2B and empty vector. (ns, nonsignificant; * *p* < 0.05; ** *p* < 0.01; *** *p* < 0.001).

The function of NS2B as an IFN antagonist was further studied in DF1 cells infected with NDV. Once again, transfection of DF1 cells with the six plasmids showed efficient expression of the six Myc-tagged NSs (Figure S5). NDV infection efficiently induced the IFN-β expression at 24 h post-infection, and overexpression of NS2B significantly inhibited the IFN-β induction (Figure 6A). It was also noted that significantly higher viral replication was detected in cells overexpressing the Myc-tagged NS2B (Figure 6B).

The effects of NS2B overexpression on the expression of IFN-inducible genes IRF7, IRF1, VIPERIN, IFIT5, and CMPK2 were also studied. DF1 cells overexpressing NS2B were infected with NDV for 12 and 24 h, respectively, and the expression levels of the induced target genes were determined by RT-qPCR. As shown in Figure 6C, overexpression of NS2B showed a 6- to 80-dold reduction at the mRNA level of IRF7, IRF1, VIPERIN, IFIT5, CMPK2, and IFN-β at 12 hpi. Interestingly, only minor to moderate reduction in the induction of IRF7, IRF1, VIPERIN, IFIT5, and CMPK2 was observed at 24 hpi (Figure 6C), probably reflecting the involvement of IFN-independent mechanisms in regulating the induction of these genes at late stages of the viral infection cycle. 

## 4. Discussion

As a newly emerging avian flavivirus in recent years, DTMUV has become a major source of infection no less than bird flu and duck plague, causing huge economic losses to the poultry industry in China. In this study, we demonstrate that the internalization and replication of DTMUV in DEFs and DF1 cells lead to the activation of several innate immunity-related signaling pathways and ISGs (Figure S6). Among them, VIPERIN, CMPK2, IFIT5, MX1, and IFN-β are the most obvious antiviral genes up-regulated, consistent with previous studies [22,31]. Further functional characterization shows that IRF1 and IRF7 play overlapping but distinct roles in limiting DTMUV replication. IRF7 induces the expression of IRF1, VIPERIN, CMPK2, IFIT5, MX1, and IFN-β, mainly through an IFN-dependent pathway, inhibiting DTMUV replication. When DTMUV-encoded NSs hijack IFN signaling, IRF1 would mediate IFN-independent mechanisms to induce the expression of VIPERIN, CMPK2, and IFIT5, thereby inhibiting the replication of DTMUV (Figure S6).

Due to the absence of IRF3 in poultry, IRF7 is the most important IRF in the regulation of type I IFNs in avian cells [32]. It has been reported that overexpression of chicken MAVS and STING in IRF7-knockout cells could not induce IFN-β activation. However, this phenotype can be reversed by the transient expression of chIRF7 [32,33]. This is consistent with our observation that overexpression of chIRF7 in IRF7-knockout cells restored the induction of IFN-β by DTMUV. In addition, overexpression of IRF7 was shown to promote the upregulation of IFIT5 induced by NDV, which in turn promoted the expression of IRF7 and NF-κB-induced genes, including IRF1 [34,35,36]. This may explain the observation in this study that expression of IRF7 in KO IFNAR1 cells promotes the expression of IFIT5 and IRF1 at the later stage of infection.

In addition to IRF3 and IRF7, IRF1 plays a critical role in regulating the expression of IFNs and ISGs [35,36]. Overexpression of duck IRF1 was previously shown to upregulate the expression of VIPERIN, IFIT5, and CMPK2 in DTMUV-infected DF1 cells [22]. Moreover, IRF1 can directly bind to and activate the VIPERIN promoter, thereby mediating the transcription of VIPERIN and inhibiting the replication of DTMUV [22]. It has been reported that IRF1 may synergistically activate IFN-β in zebrafish [37], humans [38], and ducks [39], through the downstream adaptor protein MyD88. In hepatitis E virus (HEV)-infected cells, IRF1 activates STAT1 transcription without triggering the production of IFNs and then enhances its protein expression and phosphorylation to stimulate the transcription of antiviral ISGs [40]. Vesicular stomatitis virus (VSV)-mediated VIPERIN induction occurs independently of IFN through IRF1, and the upregulation of VIPERIN transcription is sufficient to reduce VSV replication [41]. 

IRF1 may play a more dominant role in regulation of host antiviral response in the scenario when type I IFN signaling is suppressed during a viral infection [41]. It has been reported that IRF1 directly drives the constitutive expression of at least three antiviral genes, BST2, OAS2, and RNASEL, to inhibit the replication of human immunodeficiency virus (HIV), VSV, and Ebola virus [42]. In this study, we showed that DTMUV-induced IFN-β expression was severely inhibited in KO IRF7 and KO IFNAR1 DF1 cells, but overexpression of IRF1 indeed induces the expression of VIPERIN, CMPK2, and IFIT5 in these two cell lines, further supporting the conclusion that IRF1 is able to mediate IFN-independent antiviral responses.

IRF1 and IRF7 are responsible for regulating the transcription of different types of antiviral factors, establishing an antiviral state in host cell. Duck IRF1 and IRF7 were shown to activate the expression of duck IFN-β in a complementary way through the MyD88-dependent signaling pathway [43]. In macrophage-like dendritic cells, the induction of IRF1-dependent type I IFN was significantly weaker than that in virus-infected fibroblasts and plasmacytoid dendritic cells induced by IRF7, indicating that, upon effectively activating IRF7, the contribution of IRF1 may be overwhelmed by IRF7 [43]. The chicken MDA5 signaling pathway mainly relies on IRF7 to induce the expression of IFN-β [44]. However, some studies have found that overexpression of chIRF1 led to a significant up-regulation of MDA5 mRNA [45]. Therefore, chIRF1 may play a supplementary role for IRF7 in the MDA5 signaling [45]. In addition, IRF1 and 7 were found to be highly expressed in a variety of bat tissues, regulating the expression of different gene subsets. However, due to the low level of IFN induced by pathogens in bat cells, the expression of antiviral genes is mostly regulated in an IFN-independent manner [46]. In the present study, overexpression of IRF1 in KO IRF7 cells did not restore the IFN-β expression, whereas overexpression of IRF1 in KO IFNAR1 in the presence of IRF7 increased the IFN-β expression at the later stage of viral infection, supporting the dominant role of IFN-β induction by IRF1 in DTMUV-infected DF1 cells.

Similar to other viruses, DTMUV has evolved a variety of strategies to evade host antiviral defense mechanisms. DTMUV NS1 protein inhibits the RIG-like receptor signaling pathway by disrupting the interaction between RIG-I/MDA5 and MAVS [47]. NS2A and TBK1 compete with STING to inhibit IFN production and subsequent stages of IFN signaling [25]. NS2B cuts and combines duck STING to subvert the induction of IFNβ [23] and can also target MDA5 for rapid degradation [48]. NS4B significantly inhibits IFN-β and the ISRE promoter activity by competitively binding to TBK1 and STING, resulting in a decrease in TBK1 phosphorylation. Other flaviviruses have also evolved strategies to countermeasure the host antiviral responses. For example, West Nile virus (WNV) NS1 antagonizes the production of IFN-β by targeting RIG-I and MDA5 [49]. Dengue virus (DENV) NS2B3 complex has been shown to target and cleave MAVS/STING, thereby inhibiting type I IFN-mediated innate immune response [50,51]. NS2A and NS4B block RIG-I/MAVS signal transduction by inhibiting TBK1/IRF3 phosphorylation [52]. In this study, overexpression of DTMUV NS2B protein inhibited the expression of IFN-β induced by NDV and poly(I:C), confirming the IFN antagonism function of the protein.

In conclusion, this study profiles the activation of IFNs and multiple ISGs in DTMUV-infected cells through genome-wide transcriptomic analysis of chicken-derived and duck-derived cells. Functional characterization further demonstrated that IRF1 and IRF7 may regulate the expression of IFN-β and VIPERIN, as well as a number of other ISGs in a complementary manner to inhibit the replication of DTMUV. DTMUV NS2B can inhibit the induction of IFNβ induced by poly(I:C) or NDV. This study laid a foundation for further in-depth analyses of the interactions between DTMUV and the host antiviral innate immunity and the molecular pathogenesis of DTMUV.

## Data Availability

The original contributions presented in the study are publicly available. This data can be found here: https://www.ncbi.nlm.nih.gov/sra/PRJNA825282 (accessed on 11 June 2022).

## Supplementary Materials

The following supporting information can be downloaded at: https://www.mdpi.com/article/10.3390/v14071506/s1, Figure S1: Sequence analysis of IRF7-knockout (IRF7^−/−^) and IFNAR1-knockout (IFNAR1^−/−^) cells; Figure S2: The growth properties of IRF7-knockout (IRF7^−/−^) IFNAR1-knockout (IFNAR1^−/−^) cells; Figure S3: Effects of IRF7-overexpression and –knockout on upregulation of IRF7; Figure S4: Expression of IRF1 induced by overexpression of duck IRF1 in KO IRF7 and KO IFNAR1 cells; Figure S5: Determination of transfection of DTMUV NS1-NS4B in cells treated with poly (I:C) or NDV; Figure S6: Diagram outlining the overlapping and distinct roles of IRF1 and IRF7 in regulating the induction of IFN-β, VIPERIN, IFIT5 and CMPK2 as well as IRF1 and IRF7 during DTMUV infection; Figure S7: RT-qPCR verification of the top 10 genes with highest expression levels among all the up-regulated genes from the total transcriptomic data; Figure S8: ELISA assay of IFN-β in Figure 2, Figure 3, Figure 4 and Figure 5; Figure S9: Effect of IRF1 or IRF7 on MX1 in DTMUV-infected DF1; Table S1: Primers used in this study; Table S2: Differentially expressed genes in TLRs, RLRs, NLRs and hepatitis signaling pathways.
