# Supplementary material for: Avian IRF1 and IRF7 Play Overlapping and Distinct Roles in Regulating IFN-Dependent and -Independent Antiviral Responses to Duck Tembusu Virus Infection
Source: Viruses. 2022 Jul 9;14(7):1506. doi: 10.3390/v14071506 (PMC9315619; doi:10.3390/v14071506)
Supplement: Supplementary file 1 [file viruses-14-01506-s001.zip › TABLE S-2.pdf]

Supplementary Table 2

Table S2 Differentially expressed genes in TLRs, RLRs, NLRs and hepatitis signaling pathways

Pathway	Order	DF1	Log_2（FC）	DEF	Log_2（FC）
**TLR**	**1**	IFNβ	11.70	IRF7	6.17
	**2**	IFNα	6.88	IFNβ	4.72
	**3**	IRF7	5.77	IFNα	4.60
	**4**	IL12	5.70	STAT1	3.70
	**5**	IL6	5.60	TLR3	2.30
	**6**	CCL4	3.16	PIK3CA	1.62
	**7**	IL1β	3.10	FADD	1.58
	**8**	TLR3	2.90	NFKBIA	1.49
	**9**	NFKBIA	2.50	IKBKE	1.46
	**10**	MYD88	1.51	IFNAR2	1.43
**RLR**	**1**	IFNβ	11.70	IRF7	6.17
	**2**	IFNα	6.88	IFNβ	4.72
	**3**	MDA5	6.88	IFNα	4.60
	**4**	IRF7	5.77	RIG-I	4.10
	**5**	IL12	5.70	MDA5	3.80
	**6**	LGP2	4.80	MITA	3.52
	**7**	TRIM25	3.70	TRIM25	3.39
	**8**	MITA	3.14	LGP2	3.30
	**9**	NFKBIA	2.50	FADD	1.58
	**10**	NLRX1	1.82	NFKBIA	1.49
**NLR**	**1**	IFNβ	11.70	IRF7	6.17
	**2**	IFNα	6.88	GBP1	5.21
	**3**	IRF7	5.77	IFNβ	4.72
	**4**	A20	3.60	IFNα	4.60
	**5**	P2RX7	3.50	GBP2	3.76
	**6**	STING	3.13	STAT1	3.70
	**7**	IL1β	3.10	MITA	3.52
	**8**	BIRC3	3.00	RIPK2	2.80
	**9**	GBPS	2.60	TRAF5	2.78
	**10**	NFKBIA	2.50	TRPV2	2.41
**Hepatitis C**	**1**	IFNβ	11.70	IRF7	6.17
	**2**	MX	8.70	IFNβ	4.72
	**3**	VIPERIN	7.20	IFNα	4.60
	**4**	IFNα	6.88	RIG-I	4.10
	**5**	IRF7	5.77	IRF1	3.94
	**6**	PKR	3.40	MDA5	3.80
	**7**	SOCS3	3.00	STAT1	3.70
	**8**	STAT2	3.00	TLR3	2.30
	**9**	TLR3	2.90	PIK3CA	1.62
	**10**	P21	2.30	FADD	1.58
