# Supplementary material for: Avian IRF1 and IRF7 Play Overlapping and Distinct Roles in Regulating IFN-Dependent and -Independent Antiviral Responses to Duck Tembusu Virus Infection
Source: Viruses. 2022 Jul 9;14(7):1506. doi: 10.3390/v14071506 (PMC9315619; doi:10.3390/v14071506)
Supplement: Supplementary file 1 [file viruses-14-01506-s001.zip › TABLE S1.pdf]

Supplementary Table 1

Table S1 Primers used in this study

	Gene	Foward primer（5′-3′）	Reverse primer（5′-3′）
Sequences of sgRNA	IRF7（C）	GGTCGTCGTTGCACTTGGAG	CCTCCAAGTGCAACGACGACC
	IFNAR1（C）	ACCCTAATGTGGAACTACAC	TGGGATTACACCTTGATGTG
Primers for cloning	IRF7（C）	GGATCCATGGCAGCACTGGACA	CTCGAGTCAGTCTGTCTGCATGTGGT
	IRF1（D）	GGATCCATG CCCGTCTCCAG	CTCGAGTTACAACCACAGGAGA
Primers for qPCR	GAPDH（C）	GCCATCACAGCCACACAGA	TTTCCCCACAG CCTTAGCA
	VIPERIN（C）	TCGTTCTGCCTCTGCTCTCCTG	TTGTAGTTGCACTGCCTGGTGAAG
	IFIT5（C）	CACCAGCTAGGACTCTGCTACCG	CCTCCGCATACATC CTTGCCAAG
	CMPK2（C）	ATCGGTGCTGGACATCCTGGAG	GCAAGCTGG CGGAGACCTTAAC
	IRF1（C）	AAGGAGCAGGACGGCGAGATC	ACGGTGT CCAGCCAGGAGAAG
	IFN-β（C）	AGATGGCTCCCAGCTCTACA	AGTGGTTGAGCTGGTTG AGG
	IRF7（C）	ACACTCCCACAGACAGTACTGA	TGTGTGTGCCCACAGGGTTG
	DTMUV-E	CGCTGAGATGGAGGATTATGG	ACTGATTTTTGGTGG CGTG

Note：C, Chicken；D, Duck
