# Supplementary figures and images for: Avian IRF1 and IRF7 Play Overlapping and Distinct Roles in Regulating IFN-Dependent and -Independent Antiviral Responses to Duck Tembusu Virus Infection
Source: Viruses. 2022 Jul 9;14(7):1506. doi: 10.3390/v14071506 (PMC9315619; doi:10.3390/v14071506)

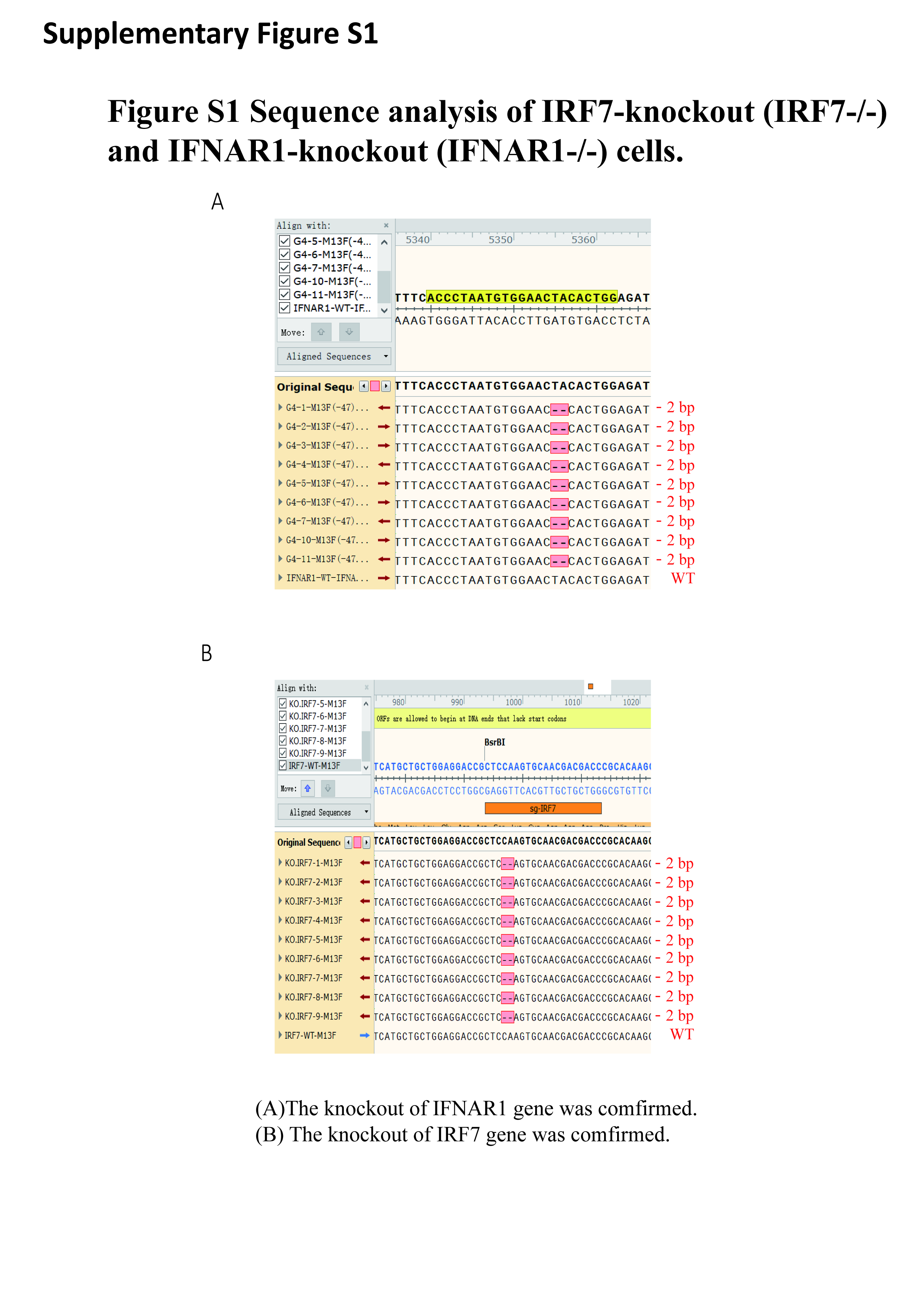

Supplement: Supplementary file 1 [file viruses-14-01506-s001.zip › FIGURE S1.tif]

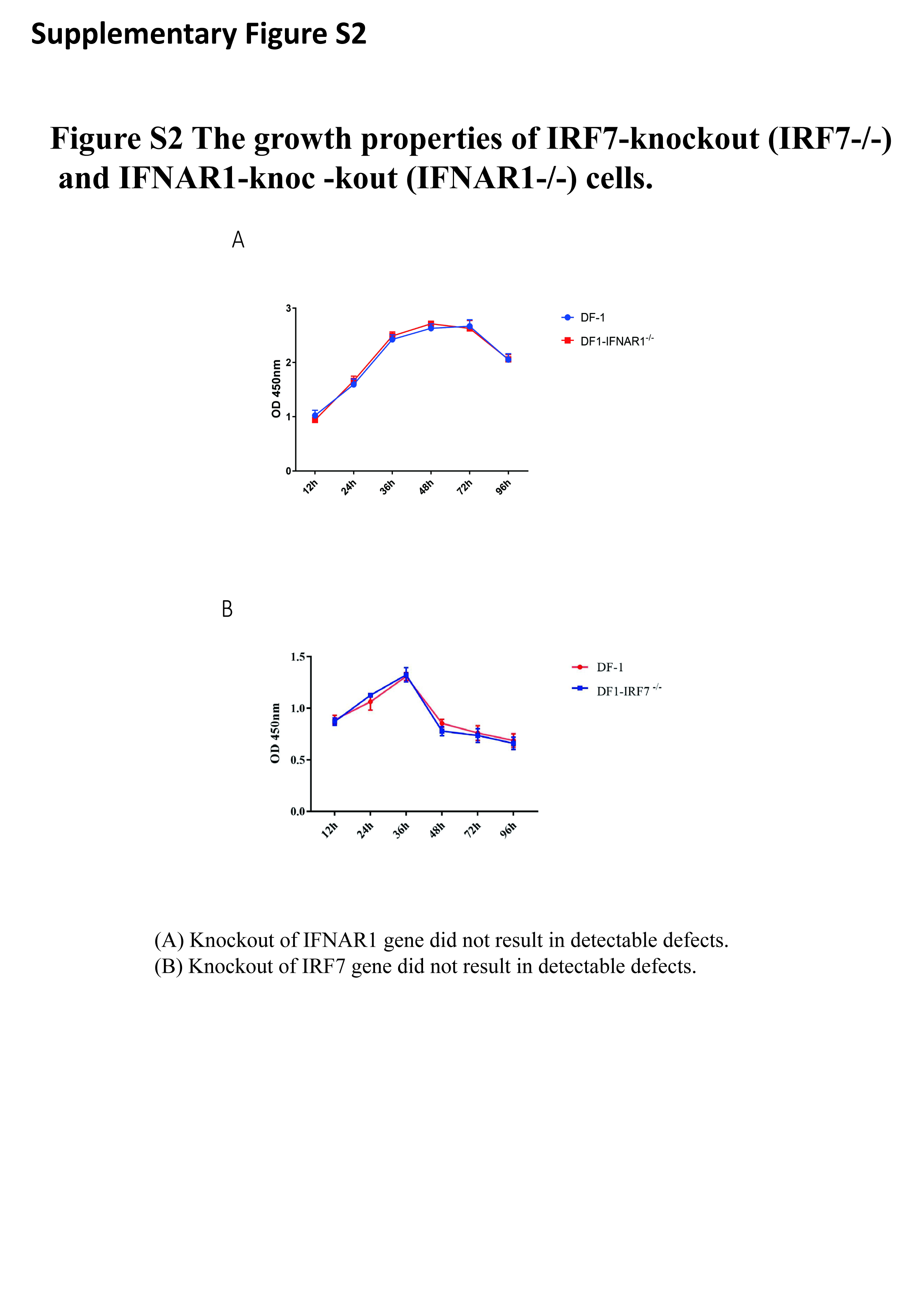

Supplement: Supplementary file 1 [file viruses-14-01506-s001.zip › FIGURE S2.tif]

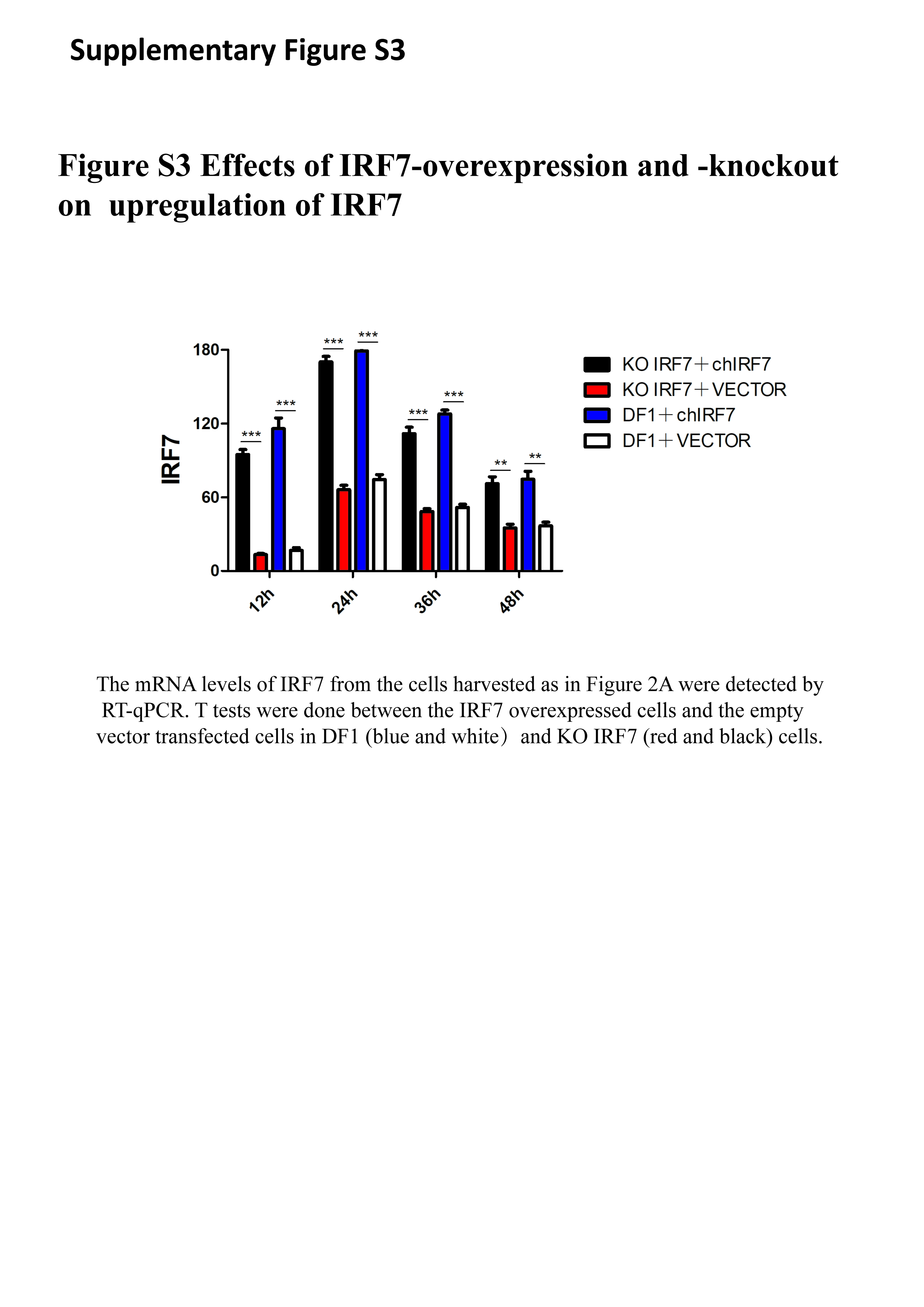

Supplement: Supplementary file 1 [file viruses-14-01506-s001.zip › FIGURE S3.tif]

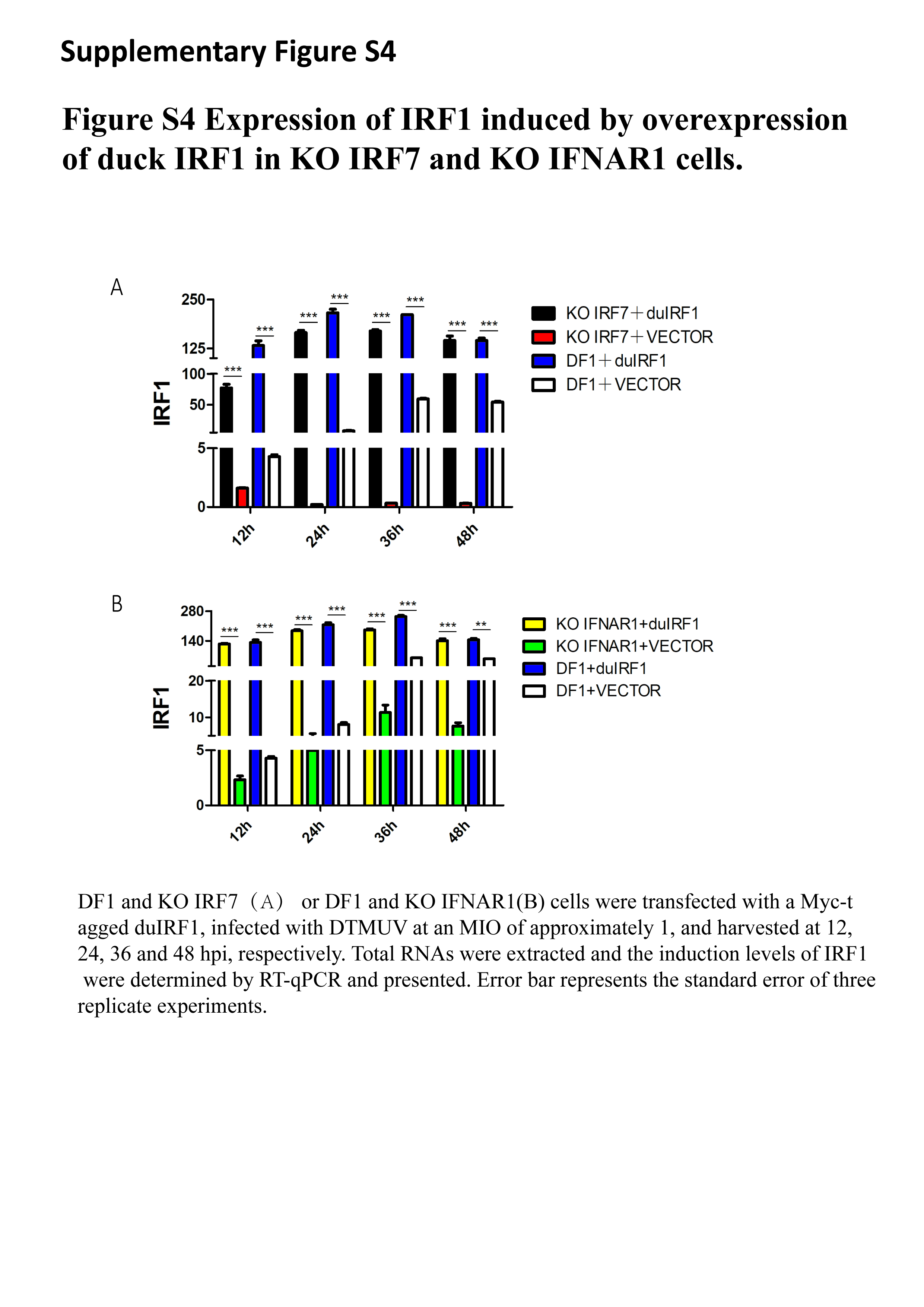

Supplement: Supplementary file 1 [file viruses-14-01506-s001.zip › FIGURE S4.tif]

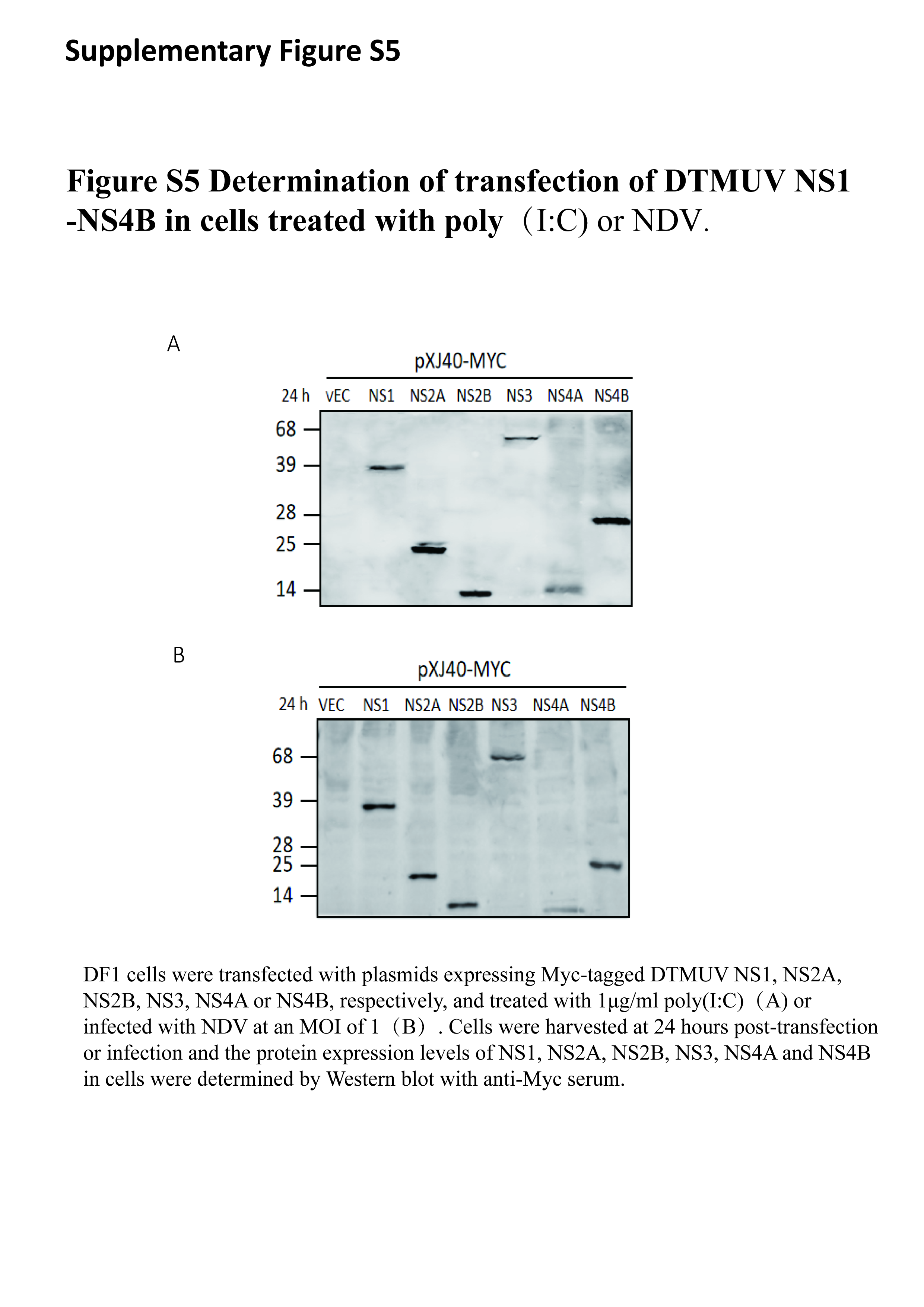

Supplement: Supplementary file 1 [file viruses-14-01506-s001.zip › FIGURE S5.tif]

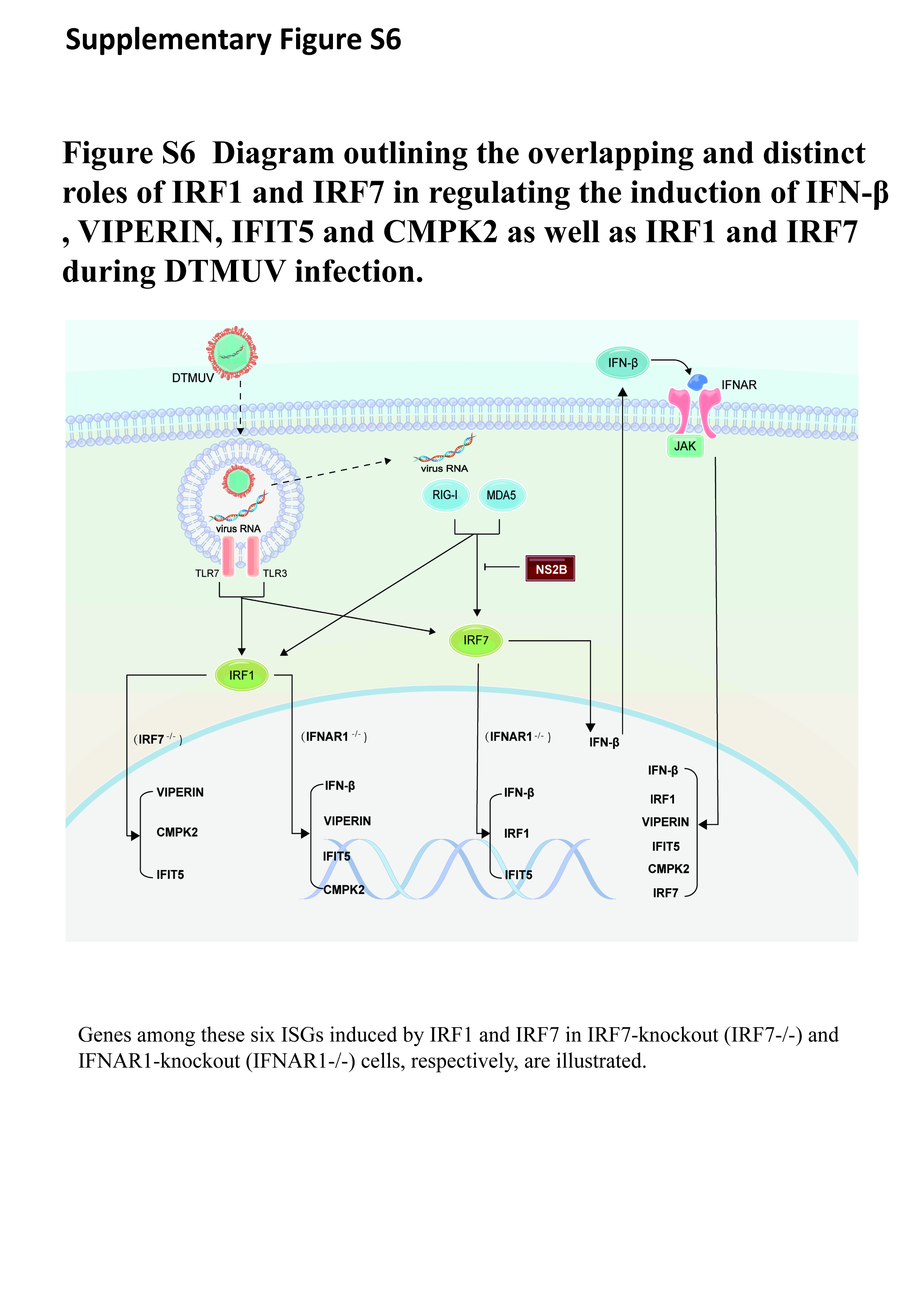

Supplement: Supplementary file 1 [file viruses-14-01506-s001.zip › FIGURE S6.tif]

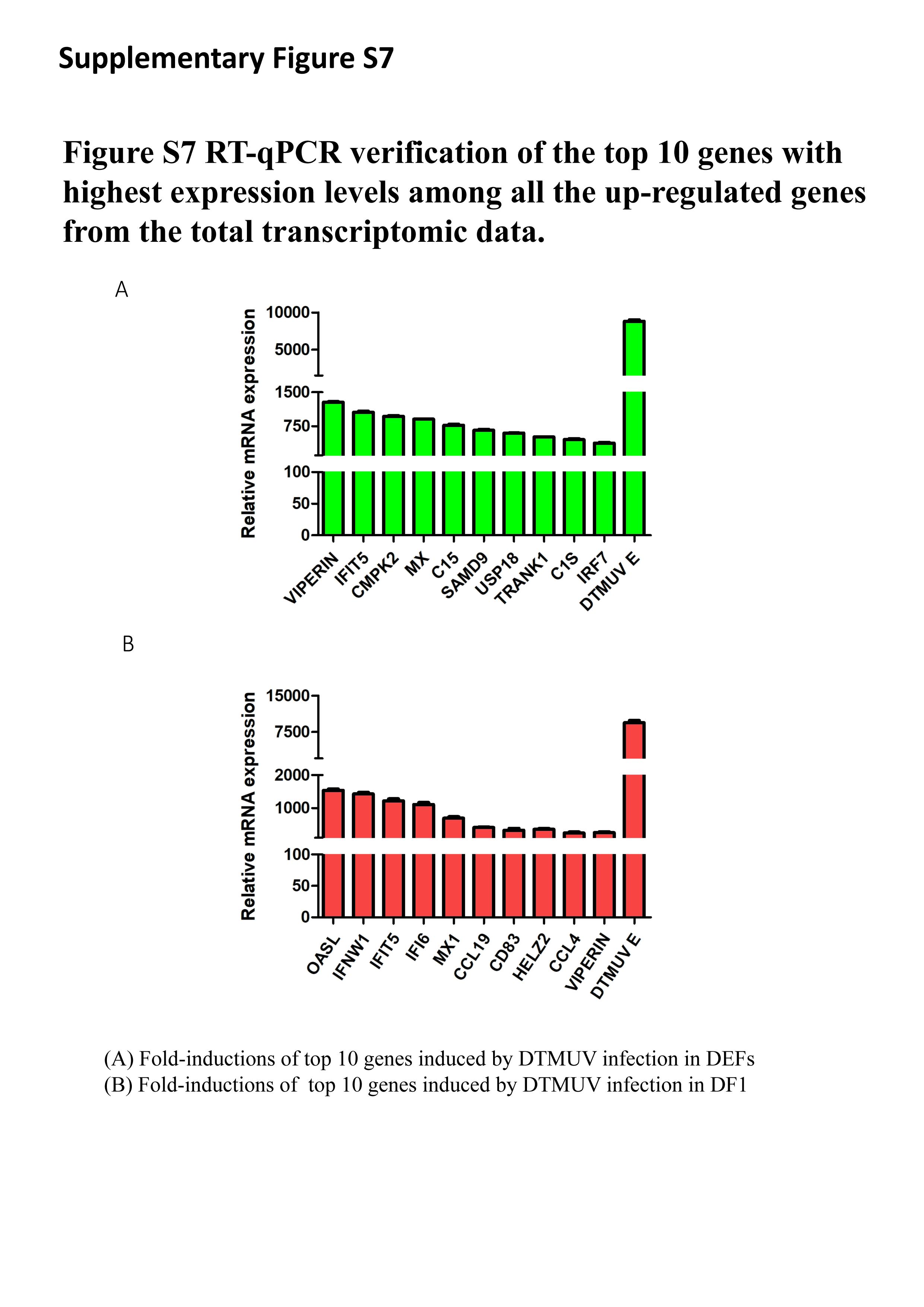

Supplement: Supplementary file 1 [file viruses-14-01506-s001.zip › FIGURE S7.tif]

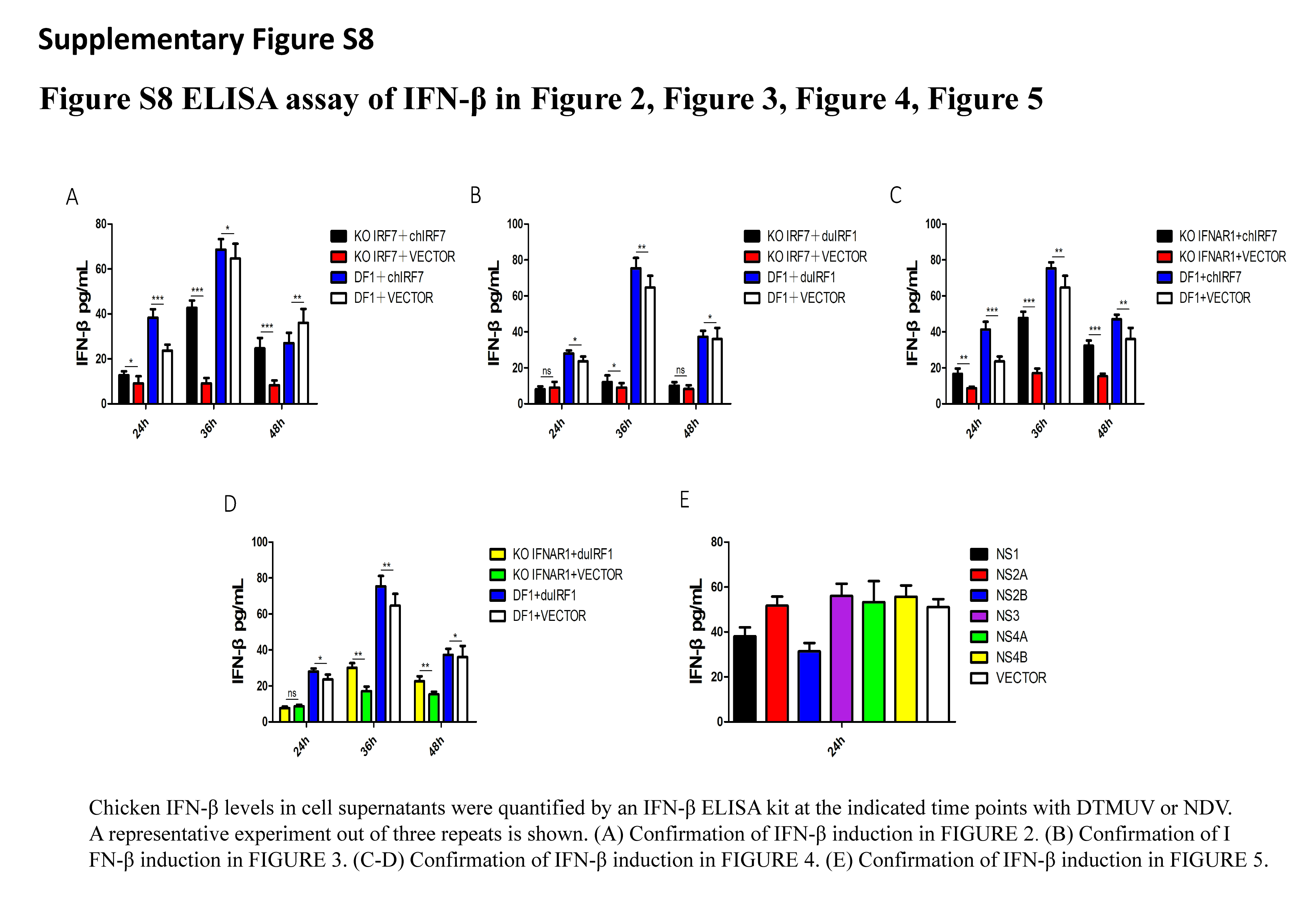

Supplement: Supplementary file 1 [file viruses-14-01506-s001.zip › Figure S8.tif]

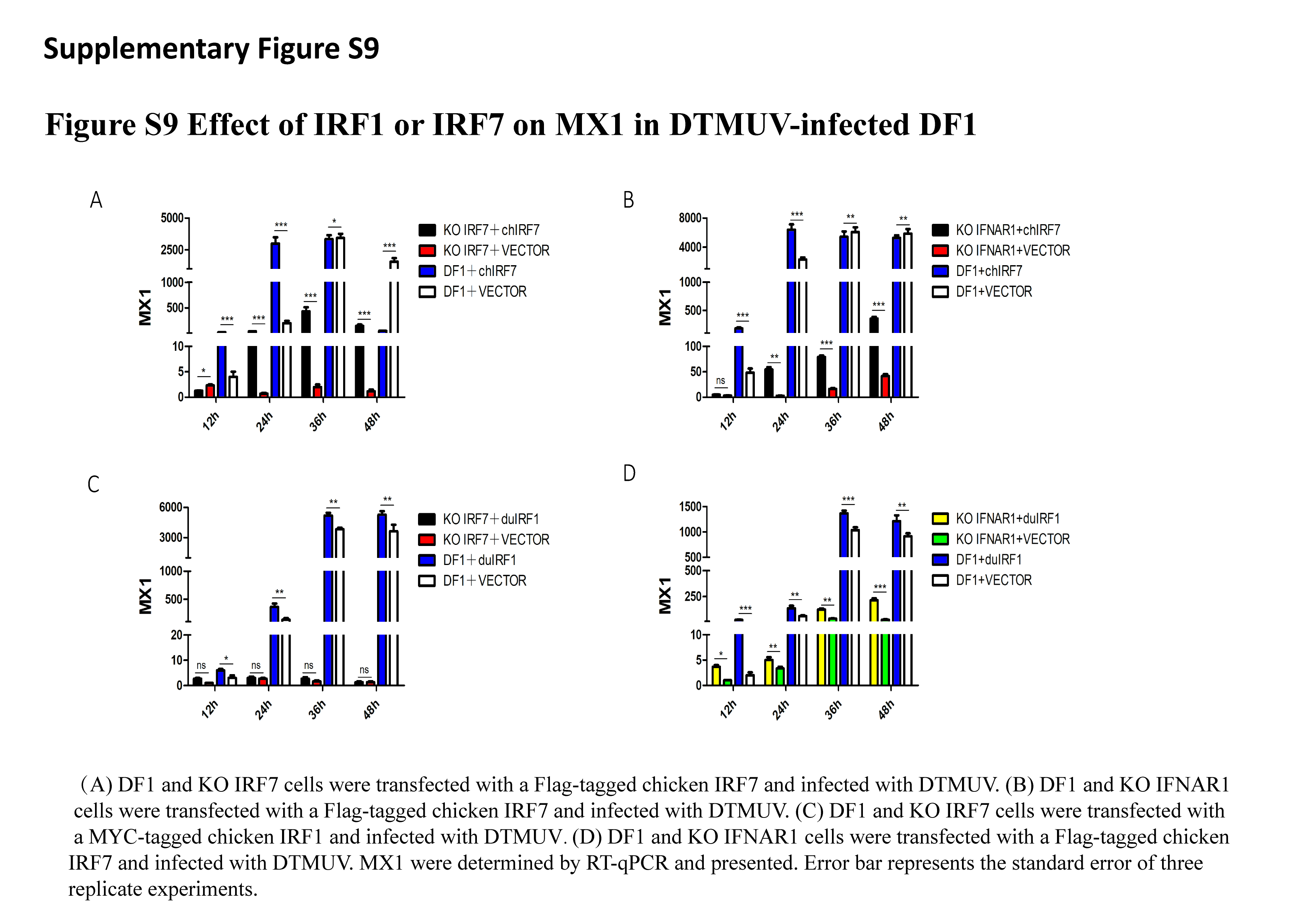

Supplement: Supplementary file 1 [file viruses-14-01506-s001.zip › Figure S9.tif]
